# Eligibility of the Systolic Blood Pressure Intervention Trial (SPRINT) to the Chinese Adults

**DOI:** 10.1155/2020/4751756

**Published:** 2020-10-17

**Authors:** Liping Chen, Yiyan Zhang, Juan Jin, Nannan Li, Dan Liu, Xinkun Zhai, Xiaodong Chen, Xiaodan Yuan

**Affiliations:** ^1^Department of Orthopaedics, Affiliated Hospital of Intergrated Traditional Chinese and Western Medicine, Nanjing University of Chinese Medicine, Jiangsu Province Academy of Traditional Chinese Medicine, Nanjing, 210028 Jiangsu, China; ^2^Department of Cardiology, Affiliated Hospital of Intergrated Traditional Chinese and Western Medicine, Nanjing University of Chinese Medicine, Jiangsu Province Academy of Traditional Chinese Medicine, Nanjing, 210028 Jiangsu, China; ^3^Department of Health Education, Affiliated Hospital of Intergrated Traditional Chinese and Western Medicine, Nanjing University of Chinese Medicine, Jiangsu Province Academy of Traditional Chinese Medicine, Nanjing, 210028 Jiangsu, China

## Abstract

**Objective:**

To explore the proportion and characteristic of Chinese adults meeting *The Systolic Blood Pressure Intervention Trial* (SPRINT) eligibility criteria and assess its generalizability.

**Method:**

Our study was based on a cross-sectional, population-based survey with a sample of 26,093 participants aged over 20 years. The SPRINT eligibility criteria were age ≥ 50 years, elevated SBP of 130 to 180 mmHg depending on the number of antihypertensive medication classes being taken, and increased cardiovascular disease (CVD) but without diabetes, history of stroke and estimated glomerular filtration rate < 20 ml/min/1.73 m^2^, or receiving dialysis.

**Results:**

Overall, we estimated that 4,036 (15.5%) participants would meet the SPRINT eligibility criteria. They were generally older, likely to be female, lower educational level, tended to be more overweight, and had higher Framingham risk score compared with overall population or subjects aged ≥ 50 years. Of participants eligible for SPRINT, most (56.2%) of them were not treated for hypertension, and 542 (13.4%) were not previously considered to have hypertension or need for antihypertension therapy. Among the 11,637 adults with hypertension, 3,494 (30.0%) would potentially benefit from treatment intensification. The most common antihypertensive medication class being taken was diuretic agents.

**Conclusion:**

A substantial percentage of Chinese subjects meet the SPRINT eligibility criteria. Further studies are needed to assess the cost-effectiveness from treatment intensification in Chinese setting.

## 1. Introduction

Results from the Systolic Blood Pressure Intervention Trial (SPRINT) indicated that compared with a standard systolic blood pressure (SBP) target of <140 mmHg, intensive treatment with a SBP target of <120 mmHg could significantly decrease the incidence of cardiovascular disease (CVD) by 25% and overall death by 27% among high-risk patients without diabetes mellitus or prior history of stroke [1]. The significant findings from this landmark study have dramatically influenced the hypertension management in Canada and the United States. The 2017 guideline of the American College of Cardiology and American Heart Association (ACC/AHA) recommends an office visit blood pressure (BP) ≥ 130/80 mmHg as the new threshold for diagnosis of hypertension, and states that the treatment goal for all hypertension patients should be lowered to <130/80 mmHg^2^. This intensive systolic treatment target was also adopted into the Hypertension Canada clinical practice guidelines [2].

Following the realease of this postive trial result and the guidelines, lively debates emerged among medical societies [3–5]. A major point of controversy is the generalizability of the findings from the trial population to patients in clinical practice [3, 6, 7]. Two studies from Canada and the United States have tried to evaluate the eligbility of SPRINT subjects to their respective border popualtion [8, 9]. Both studies have shown that a substantial percentage of adults meet the eligibility criteria for SPRINT with a lower eligbility rate among Canadian adults (5.2% vs. 7.6% in American population).

However, it is not known about the eligibility of the SPRINT intensive SBP treatment strategy among Chinese adults, whose hypertension drug use patterns and disease epidemiologic factors differ from other population (e.g., American or Canadian) [10–13]. Thus, the purpose of this study was to examine the percentage of Chinese adults who were potentially eligible for the SPRINT criteria and further determine the characteristics of these subjects.

## 2. Methods

### 2.1. Study Population

A population-based cross-sectional survey was carried out to investigate the risk factors for cardiovascular disease in Nanjing, Jiangsu Province, south of China. An initial sample of 5,824 participants was obtained using a random cluster sampling among the residents of six communities in 2011. In each community, one street district or township was randomly selected. All households within the selected street or town were included with only one participant aged ≥ 20 years selected from each household, without replacement. The detailed study design has been described elsewhere [14]. In 2013, we further selected 21 communities and followed the identical survey protocol as at the initial stage. In current analysis, we included 26,093 subjects after excluding 43 subjects without BP measurements. All participants provided informed consent, and the study was approved by the institutional review boards of Jiangsu Province Hospital on Integration of Chinese and Western Medicine.

### 2.2. Data Collection

A face-to-face interview was conducted by trained research staffs. Information was collected through a standard questionnaire including age, sex, education, cigarette smoking, physical activity, a prior diagnosis of disease including hypertension, diabetes, myocardial infarction, coronary heart disease (CHD), stroke, and receipt of dialysis in the past 12 months. Weight (to the nearest 0.1 kg) and height (to the nearest 0.1 cm) were measured by using calibrated instruments without shoes. Body mass index (BMI, weight divided height square) was calculated. Blood specimens were processed at the examination center. Total cholesterol (TC), high-density lipoprotein cholesterol (HDL-C), and serum creatinine were measured by automated analyser (Olympus AU600 autoanalyser (Olympus Optical, Tokyo, Japan).

Educational level was classed as none, primary, secondary/high school/higher secondary, and trade school/college/university. Physical activity was assessed by the Long-Form International Physical Activity Questionnaire and was classed as low, moderate, and high by the tertile of metabolic equivalent of task- (MET-) minutes per week. The estimated glomerular filtration rate (eGFR, in ml/min/1.73 m^2^) was calculated using the Modification of Diet in Renal Disease equation [15].

### 2.3. Blood Pressure Measurement and Hypertension Definition

For each participant, we measured blood pressure three times on their right upper arm after 5 min of rest in a seated position with the use of the Omron HEM-757 automatic digital monitor (Omron Healthcare) attended by the examiner. Three BP measurements were obtained at 30-second interval, and the mean of the two closest recorded blood pressure measurements was used. Hypertension was defined as an average SBP of at least 140 mmHg or an average diastolic blood pressure (DBP) of at least 90 mmHg, or self-reported use of an antihypertensive drug in the past 2 weeks [16]. Treated hypertension was defined by self-reported use of medication to lower BP with 1 or more classes of antihypertension medication.

### 2.4. Medications

Participants were asked about their prescription medications taken in the past two weeks. Medication names were recorded and coded into three groups of antihypertensive medication: (1) western medicine including angiotensin-converting enzyme inhibitors (ACEIs), angiotensin receptor blockers, beta-blockers, calcium-channel blocker, diuretic agents, and others (including alpha blockers, aldosterone receptor antagonists, central-acting agents, and direct acting vasodilators); (2) Traditional Chinese medicine and West medicine compound; and (3) traditional medicine only (not considered an antihypertensive medication class while setting SBP criteria).

### 2.5. SPRINT Eligibility

**Figure 1:**
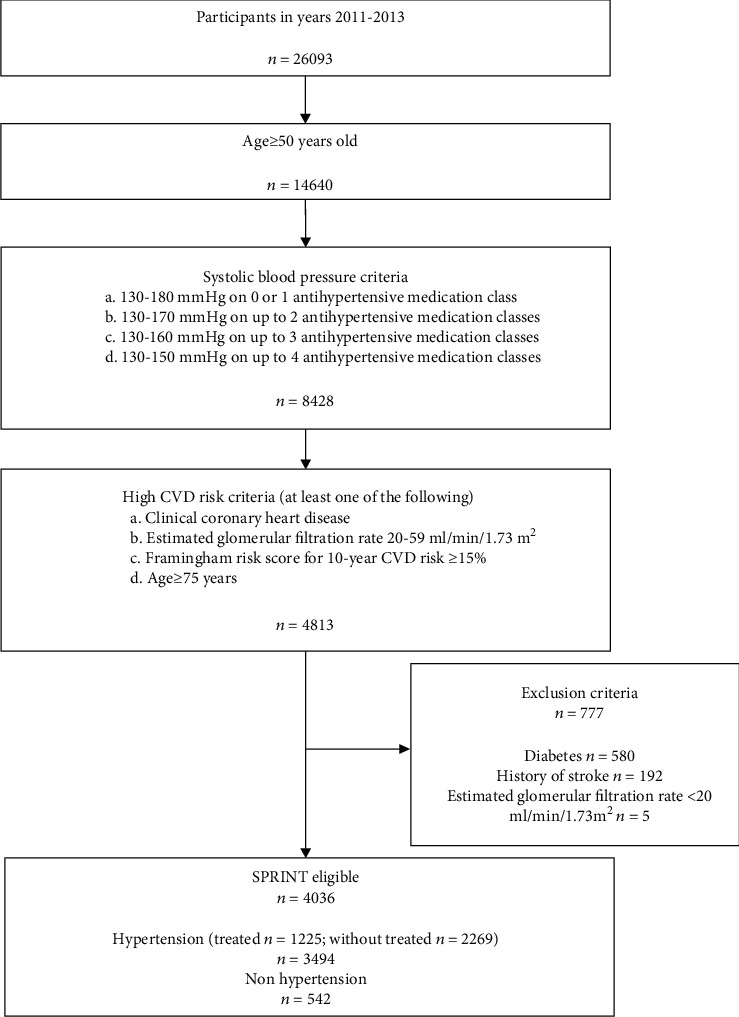
Flow chart showing the eligibility criteria for SPRINT applied to present study.

A multistep algorithm was used to determine the potential eligibility for SPRINT (Figure 1). The population was included to meet all the following criteria: an age of at least 50 years, an elevated SBP (between 130 and 180 mmHg using 0 or 1 antihypertensive medication, 130-170 mmHg using up to 2 medications, 130-160 mmHg using up to 3 medications, or 130 to 150 mmHg using up to 4 medications), and an increased risk of cardiovascular events. Increased cardiovascular risk was defined by one or more of the following: established clinical CHD; an estimated eGFR of 20 to 59 ml per minute per 1.73 m^2^; a 10-year risk of cardiovascular disease of 15% or greater on the basis of the Framingham risk score; or an age of 75 years or older. Respondents with diabetes (self-report), stroke (self-report), or an estimated eGFR less than 20 ml per minute per 1.73 m^2^ were excluded.

### 2.6. Statistical Analysis

We calculated the number and proportion of subjects meeting eligibility criteria for SPRINT, as well as each individual component. Descriptive statistics for demographic and clinical characteristics were reported for overall population, for subjects meeting SPRINT eligibility, and for those hypertensive subjects. Further, we determined the characteristics of those meeting SPRINT eligibility by their hypertension status (i.e., treated, untreated, and previously not considered to have hypertension). For comparison, we also calculated the number and proportion of antihypertensive medication classes taken by all hypertension and SPRINT-eligible hypertension adults. All analysis performed using Stata 14 (Stata Corp, College Station, Texas).

## 3. Results

The characteristics of all participants meeting each sequential SPRINT eligibility criteria are presented in Table 1. Of the overall 26,093 participants, 14,640 (56.1%) were 50 years of age or older, 8,428 (32.3%) met the SBP criteria, 4,813 (18.4%) also were additionally at high CVD risk, and finally 4,036 (15.5%) met all eligibility criteria for SPRINT. Participants who were eligible for SPRINT were generally older (mean age, 61.4 vs. 58.5), female (69.3% vs. 58.6%), had higher mean total cholesterol (194.4 vs. 178.4 mg/dl) and lower mean glomerular filtration rate (66.4 vs. 77.0 ml/min/1.73 m^2^), and were more likely to be overweight (BMI ≥ 25 kg/m^2^, 51.4% vs. 40.3%). SPRINT-eligible adults also tended to have an elevated Framingham risk score ≥ 10% compared with those not eligible. Those who met the SPRINT eligibility criteria generally had higher SBP (152 vs. 134 mmHg) and DBP (89 vs. 83 mmHg) levels compared with the overall study population. These findings are also applied to the population aged ≥ 50 years.

Of the 4,036 participants who were eligible for SPRINT, 542 (13.4%) were not considered to have preexisting hypertension defined by self-report, BP ≥ 140/90 mmHg, and/or use of any antihypertensive medicine, 1,225 (30.4%) had treated hypertension, and 2,269 (56.2%) were not treated for hypertension. The characteristics of SPRINT-eligible treated hypertension, untreated hypertension, and not previously considered to have hypertension are shown in Table 2. In general, those subjects with no hypertension were majority at moderate risk of CVD (62.2%) with a Framingham risk score of 10%-20%, despite a lower prevalence of overweight (39.7%) in comparison with those hypertensive subjects.

Additionally, the proportion of Chinese hypertension who met SPRINT eligibility criteria according to individual criteria of SPRINT eligibility was estimated (Table S1). Among the 11,637 hypertension participants, 8,231 (70.7%) were 50 years of age or older, 6,656 (57.2%) met the SBP criteria, 4,188 (36.0%) were additionally at high CVD risk, and finally 3,094 (26.6%) met the SPRINT eligibility criteria.

From Table 3, we can find that the majority (64.9%) of SPRINT-eligible hypertension participants did not treat hypertension, 1,004 (28.7%) of them had one class of antihypertensive medication identified, 203 (5.8%) of them had two classes of antihypertensive medication identified, and only 19 (0.6%) of them had three or more classes of antihypertensive medication identified. Among 1,225 (35.1%) treated hypertension who met the SPRINT eligibility criteria, the most common antihypertensive medication classes being taken were diuretic agents (45.7%) and followed by calcium-channel blocker (34.2%).

## 4. Discussion

Among the 26,093 participants, approximately 15.5% adults would meet eligibility criteria for the SPRINT study and potentially benefit from intensive SBP lowering to a goal of <120 mmHg. Most of SPRINT-eligible adults (56.2%) were not treated hypertension, 1,225 (30.3%) participants were taking antihypertensive medication, and 542 (13.4%) individuals would be reclassified to be treated with blood pressure lowering therapy, although not previously considered to have hypertension. Nearly thirty percent of hypertensive adults would potentially qualify for treatment intensification.

It can be found that the proportion of Chinese (15.5%) who met the SPRINT eligibility criteria in our present sample was higher than the US adults (7.6%) and Canadian (5.2%). Reasons for the low percentage of US adults meeting the SPRINT eligibility criteria included a high percentage of US adults <50 years of age with SBP ≥ 130 mmHg and elder participants in our study sample compared with NHANES participants [13]. Additionally, our study found that SPRINT-eligible adults were generally with older age, lower education, more obesity, higher Framingham risk score, and higher BP level compared with overall population, which is generally consistent with the findings from Canadian and American population [8, 9]. A possible explanation is that the SPRINT study enrolled participants with age ≥ 50 years and at high risk for CVD [1]. However, disparities in eligibility for SPRINT between Chinese population and US adults were also presented by sex. In the US, males were more likely to meet the SPRINT eligibility criteria compared with females. On the contrary, females accounted for a major proportion in China.

The SPRINT treatment for intensive group begins with two or three drugs therapy using a combination of a thiazide-type diuretic, and/or an ACEI or ARB and/or calcium-channel blocker [1]. In our analysis, only 28.4% of hypertension adults and 35.1% of hypertension adults who met the SPRINT eligibility criteria were taking antihypertensive medication. Among those two groups, the most common antihypertensive medication class being taken is diuretic agent. However, it was ACE inhibitors in the US [9]. This difference may be attributable to the lower cost of diuretic than other antihypertensive medications in China or different treatment guidelines between the two countries [16–18].

Despite greater cardiovascular protection with intensive blood pressure lowering, achieving SPRINT-defined blood pressure goals might be challenging in China because the target blood pressure of <120 mmHg was not met among more than one-half of the participants in our SPRINT-eligible adults. Furthermore, it is expected that a more aggressive and time-consuming approach is needed to achieve SPRINT SBP goals, which would require more economic investment on hypertension management in China. Although the SPRINT investigators have found that treating to an SBP goal of <120 mmHg compared with <140 mmHg may be cost effective (at ≤$100,000 per quality-adjusted life year gained) [19] and supported by another two studies [20, 21], a comprehensive study is needed to assess the potential clinical and cost implications from treatment intensification in Chinese setting.

Our study has several potential limitations. Firstly, our study sample is not from a nationally representative survey; therefore, we could not produce national estimates of Chinese adults who might be eligible for SPRINT. Secondly, not all of the SPRINT eligibility criteria were collected in our study while designing our study, such as polycystic kidney disease and symptomatic heart failure. This may overestimate the proportion of those meeting SPRINT's intensive treatment. Thirdly, the presence of comorbidities and use of medication was gathered using a questionnaire and was not verified with medical records. This might have recall bias and led to some misclassification, which may overestimate or underestimate our estimates. Lastly, because of our study was a cross-sectional study; we cannot support the idea that individuals may benefit from lowering SBP to <120 mmHg using antihypertensive in Chinese adults. However, to the best of our knowledge, this is the first study to explore the generalizability of SPRINT results to Chinese adult population. Our findings would have important implications for policies aiming at enhancing prevention and control of hypertension in China.

## 5. Conclusion

Adoption of intensive SBP lowering to <120 mmHg in SPRINT-eligible high-risk individuals would increase the proportion of Chinese adults receiving BP treatment initiation or intensification. Further studies are needed to assess the cost-effectiveness from treatment intensification in Chinese setting.

## Figures and Tables

**Table 1:** Overall participants meeting each sequential SPRINT eligibility criterion.

Overall participants, *N*	Overall	Age ≥ 50 y	+SBP criteria^1^	+High CVD risk^2^	+Exclusion Criteria^3^
	26093	14640	8428	4813	4036
Socioeconomic background					
Age (years)	51.22 ± 9.89	58.49 ± 6.22	59.03 ± 6.15	61.34 ± 6.03	61.35 ± 6.09
Gender (male, %)	10,881 (41.7%)	6,201 (42.4%)	3,636 (43.1%)	1,555 (32.3%)	1,281 (31.7%)
Educational level					
None	1,698 (6.5%)	1,421 (9.7%)	829 (9.9%)	568 (11.9%)	490 (12.2%)
Primary	6,275 (24.1%)	4,651 (31.9%)	2,779 (33.1%)	1,675 (34.9%)	1,434 (35.7%)
Secondary/high school/higher secondary	14,311 (55.0%)	6,602 (45.3%)	3,694 (44.0%)	1,890 (39.4%)	1,559 (38.8%)
Trade school/college/university	3,729 (14.4%)	1,911 (13.1%)	1,098 (13.1%)	662 (13.8%)	536 (13.3%)
Risk factors of hypertension					
Heart rate, beats/min	73.76 ± 10.74	73.37 ± 10.97	73.57 ± 11.08	73.84 ± 11.18	73.65 ± 11.18
Total cholesterol, mg/dl	178.40 ± 36.60	185.56 ± 36.84	187.23 ± 36.75	194.42 ± 37.97	194.41 ± 37.26
High-density lipoprotein, mg/dl	51.75 ± 12.13	53.13 ± 12.07	53.13 ± 12.17	52.90 ± 11.97	52.94 ± 12.06
Estimated glomerular filtration rate, ml/min/1.73 m^2^	77.03 ± 16.61	72.08 ± 15.32	71.37 ± 15.26	66.43 ± 14.24	66.40 ± 14.01
Current smoking	6,254 (24.3%)	3,442 (23.8%)	1,896 (22.7%)	1,033 (21.9%)	900 (22.8%)
BMI ≥ 25 kg/m^2^	10,516 (40.3%)	6,217 (42.5%)	3,953 (46.9%)	2,475 (51.4%)	2,066 (51.2%)
Framingham risk score	10.31 ± 9.53	14.86 ± 10.33	16.84 ± 9.45	21.95 ± 9.62	20.42 ± 8.11
<10%	16,219 (63.9%)	5,686 (39.9%)	1,955 (23.5%)	272 (5.8%)	263 (6.7%)
10%-20%	5,967 (23.5%)	5,435 (38.1%)	4,075 (49.1%)	2,143 (45.7%)	1,960 (49.9%)
>20%	3,189 (12.6%)	3,141 (22.0%)	2,274 (27.4%)	2,274 (48.5%)	1,704 (43.4%)
Physical activity					
Low	3,203 (12.8%)	1,673 (11.9%)	1,003 (12.4%)	577 (12.5%)	495 (12.7%)
Moderate	10,960 (43.8%)	6,280 (44.7%)	3,600 (44.4%)	2,127 (45.9%)	1,804 (46.3%)
High	10,857 (43.4%)	6,088 (43.4%)	3,498 (43.2%)	1,928 (41.6%)	1,600 (41.0%)
Blood pressure measurement					
SBP (mmHg)	133.63 ± 21.93	139.41 ± 22.62	148.11 ± 13.18	151.65 ± 13.67	151.88 ± 13.69
130-139 mmHg	4,607 (17.7%)	2,692 (18.4%)	2,692 (31.9%)	1,111 (23.1%)	910 (22.6%)
≥140 mmHg	9,110 (34.9%)	6,688 (45.7%)	5,736 (68.1%)	3,702 (76.9%)	3,126 (77.4%)
DBP (mmHg)	82.62 ± 12.04	84.10 ± 12.02	88.01 ± 9.84	88.60 ± 10.31	88.92 ± 10.33
80-89 mmHg	8,277 (31.7%)	4,812 (32.9%)	3,329 (39.5%)	1,754 (36.4%)	1,455 (36.1%)
≥90 mmHg	7,044 (27.0%)	4,579 (31.3%)	3,590 (42.6%)	2,221 (46.2%)	1,915 (47.5%)

Reported as mean ± SD or number (%). Abbreviations: CVD: cardiovascular disease. DBP: diastolic blood pressure. SBP: systolic blood pressure. SPRINT: Systolic Blood Pressure Intervention Trial. ^1^SBP criteria include 130-180 mmHg on 0 or 1 antihypertensive medication class; 130-170 mmHg on 2 classes; 130-160 mmHg on 3 classes; and 130-150 mmHg on 4 classes. ^2^High CVD risk includes history of CHD, eGFR of 20-59 ml/min/1.73 m^2^, 10-year risk for CVD ≥ 15%, and age ≥ 75 years. ^3^Exclusion criteria include diabetes, history of stroke, and eGFR < 20 − 59 ml/min/1.73 m^2^.

**Table 2:** Participants meeting SPRINT eligibility criterion by hypertension status.

	Hypertension		No hypertension
	Treated^1^	Untreated	
Sample, *N*	1,225	2,269	542
Socioeconomic background			
Age (years)	60.96 ± 5.91	61.50 ± 6.06	61.61 ± 6.61
Gender (male, %)	380 (31.0%)	775 (34.2%)	126 (23.3%)
Educational level			
None	132 (10.8%)	298 (13.2%)	60 (11.1%)
Primary	405 (33.1%)	842 (37.3%)	187 (34.8%)
Secondary/high school/higher secondary	491 (40.2%)	861 (38.1%)	207 (38.5%)
Trade school/college/university	194 (15.9%)	258 (11.4%)	84 (15.6%)
Risk factors of hypertension			
Heart rate, beats/min	72.26 ± 11.19	74.57 ± 11.32	72.92 ± 10.17
Total cholesterol, mg/dl	195.40 ± 36.70	193.86 ± 36.74	194.50 ± 40.55
High-density lipoprotein, mg/dl	52.96 ± 11.23	53.05 ± 12.50	52.42 ± 12.00
Estimated glomerular filtration rate, ml/min/1.73 m^2^	66.35 ± 13.77	67.02 ± 14.14	63.94 ± 13.76
Current smoking	214 (17.6%)	591 (26.6%)	95 (18.3%)
BMI ≥ 25 kg/m^2^	724 (59.1%)	1,127 (49.7%)	215 (39.7%)
Framingham risk score	23.76 ± 9.04	19.75 ± 7.14	15.38 ± 6.20
<10%	31 (2.6%)	125 (5.7%)	107 (20.9%)
10%-20%	465 (38.3%)	1,176 (53.4%)	319 (62.2%)
>20%	717 (59.1%)	900 (40.9%)	87 (16.9%)
Physical activity			
Low	125 (10.4%)	309 (14.1%)	61 (12.0%)
Moderate	571 (47.6%)	1,008 (46.0%)	225 (44.1%)
High	503 (42.0%)	873 (39.9%)	224 (43.9%)
Blood pressure measurement			
SBP(mmHg)	153.91 ± 13.15	155.04 ± 12.27	134.12 ± 2.89
130-139 mmHg	203 (16.6%)	165 (7.3%)	542 (100%)
≥140 mmHg	1,022 (83.4%)	2,104 (92.7%)	0
DBP(mmHg)	90.08 ± 10.05	90.48 ± 10.15	79.75 ± 6.22
80-89 mmHg	426 (34.8%)	703 (31.0%)	326 (60.2%)
≥90 mmHg	640 (52.2%)	1,275 (56.2%)	0

Reported as mean ± SD or number (%). Abbreviations: DBP: diastolic blood pressure. SBP: systolic blood pressure. SPRINT: Systolic Blood Pressure Intervention Trial. ^1^Treated hypertension was defined by self-reported use of antihypertensive dedication with 1 or more classes.

**Table 3:** Antihypertensive medication usage among hypertensive participants by overall and those eligible for SPRINT.

	Overall	SPRINT eligible
Hypertension		
Sample, *N*	11,637	3,494
Number of classes^1^		
0	8,336 (71.6%)	2,268 (64.9%)
1	2,614 (22.5%)	1,004 (28.7%)
2	611 (5.2%)	203 (5.8%)
≥3	76 (0.7%)	19 (0.6%)
Treated hypertension		
Sample, *N*	3,301	1,225
Western medicine		
ACE inhibitor	566 (17.2%)	170 (13.9%)
Angiotensin receptor blocker	29 (0.9%)	11 (0.9%)
Beta-blocker	240 (7.3%)	88 (7.2%)
Calcium-channel blocker	1,197 (36.3%)	419 (34.2%)
Diuretic	1,443 (43.7%)	560 (45.7%)
Others^2^	115(3.5%)	33(2.7%)
TCM-West compound	441 (13.4%)	173 (14.1%)
TCM only^3^	56(1.7%)	16(1.3%)

Reported as mean ± SD or number (%). Abbreviations: ACE: angiotensin-converting enzyme. SPRINT: Systolic Blood Pressure Intervention Trial. TCM: Traditional Chinese medicine. ^1^Number of antihypertensive medication classes included ACE inhibitors, alpha blockers, aldosterone receptor antagonists, angiotensin receptor-blockers, beta-blocker, calcium-channel blockers, central-acting agents, diuretic, renin inhibitors, direct acting vasodilators, and TCM-West compound. ^2^Others included alpha blockers, aldosterone receptor antagonists, central-acting agents, direct acting vasodilators, and TCM-West compound. ^3^TCM only group was not considered an antihypertensive medication class when setting SBP criteria.

## Data Availability

Supportive data are supplied in the appendix. Other data that support the findings of this study are available from the corresponding author upon reasonable request.

## Supplementary Materials

Supplementary materials Table S1: hypertension participants meeting each sequential SPRINT eligibility criterion.
