# Supplementary material for: Eligibility of the Systolic Blood Pressure Intervention Trial (SPRINT) to the Chinese Adults
Source: Biomed Res Int. 2020 Oct 17;2020:4751756. doi: 10.1155/2020/4751756 (PMC7591957; doi:10.1155/2020/4751756)
Supplement: Supplementary materials — Table S1: hypertension participants meeting each sequential SPRINT eligibility criterion. [file 4751756.f1.docx]

| **Supplementary table 1.** **hypertension participants meeting each sequential SPRINT eligibility criterion** | | | | | |
| --- | --- | --- | --- | --- | --- |
|  | Overall | Age≥50y | +SBP criteria^1^ | +High CVD risk^2^ | +Exclusion Criteria^3^ |
| **N** | 11637 | 8231 | 6656 | 4188 | 3494 |
| ***Socio-economic background*** |  |  |  |  |  |
| Age(Years) | 54.59±9.36 | 59.34±6.23 | 59.26±6.17 | 61.33±5.95 | 61.31±6.01 |
| Gender (male, %) | 5183(44.5%) | 3526(42.8%) | 2865(43.0%) | 1399(33.4%) | 1155(33.1%) |
| Educational level |  |  |  |  |  |
| None | 958(8.3%) | 872(10.6%) | 680(10.2%) | 503(12.1%) | 430(12.4%) |
| Primary | 3307(28.5%) | 2774(33.8%) | 2216(33.4%) | 1466(35.1%) | 1247(35.8%) |
| Secondary/High School/Higher secondary | 5795(50.0%) | 3509(42.8%) | 2876(43.4%) | 1645(39.4%) | 1352(38.8%) |
| Trade School/College/University | 11537(13.2%) | 1046(12.8%) | 860(13.0%) | 560(13.4%) | 452(13.0%) |
| ***Risk factors of hypertension*** |  |  |  |  |  |
| Heart rate, beats/min | 74.58±11.46 | 73.96±11.46 | 73.83±11.30 | 73.91±11.30 | 73.76±11.32 |
| Total cholesterol, mg/dl | 184.69±36.81 | 187.99±36.64 | 187.88±36.62 | 194.39±37.31 | 194.40±36.73 |
| High-density lipoprotein, mg/dl | 52.51±12.32 | 53.29±12.28 | 53.11±12.26 | 53.00±11.99 | 53.02±12.07 |
| Estimated glomerular filtration rate, ml/min/1.73m^2^ | 74.88±17.12 | 71.19±15.73 | 70.91±15.39 | 66.70±14.27 | 66.78±14.02 |
| Current smoking | 2769(24.1%) | 1849(22.7%) | 1468(22.3%) | 919(22.3%) | 805(23.4%) |
| BMI≥25 | 6075(52.2%) | 4186(50.9%) | 3309(49.7%) | 2225(53.1%) | 1851(53.0%) |
| Framingham risk score | 15.74±11.11 | 19.23±11.12 | 18.24±9.75 | 22.76±9.68 | 21.17±8.10 |
| <10% | 4083(36.5%) | 1399(17.6%) | 1117(17.0%) | 163(4.0%) | 156(4.6%) |
| 10%-20% | 4187(37.5%) | 3708(46.5%) | 3301(50.3%) | 1787(43.6%) | 1641(48.1%) |
| >20% | 2909(26.0%) | 2864(35.9%) | 2145(32.7%) | 2145(52.4%) | 1617(47.4%) |
| Physical activity |  |  |  |  |  |
| Low | 1468(13.1%) | 986(12.5%) | 796(12.4%) | 508(12.5%) | 434(12.8%) |
| Moderate | 4933(44.1%) | 3547(44.8%) | 2873(44.8%) | 1867(46.2%) | 1579(46.6%) |
| High | 4777(42.8%) | 3376(42.7%) | 2740(42.8%) | 1670(41.3%) | 1376(40.6%) |
| ***Blood pressure measurement*** |  |  |  |  |  |
| SBP(mmHg) | 151.71±18.84 | 153.58±19.27 | 151.88±12.25 | 154.27±12.68 | 154.64±12.59 |
| 130-139 mmHg | 1533(13.2%) | 920(11.2%) | 920(13.8%) | 486(11.6%) | 368(10.5%) |
| ≥140 mmHg | 9110(78.3%) | 6688(81.3%) | 5736(86.2%) | 3702(88.4%) | 3126(89.5%) |
| DBP(mmHg) | 91.44±10.88 | 90.57±10.98 | 90.03±9.69 | 89.91±10.15 | 90.34±10.11 |
| 80-89 mmHg | 3210(27.6%) | 2488(30.2%) | 2209(33.2%) | 1381(33.0%) | 1129(32.3%) |
| ≥90 mmHg | 7044(60.5%) | 4579(55.6%) | 3590(53.9%) | 2221(53.0%) | 1915(54.8%) |
